# The Role and Therapeutic Potential of Pyroptosis in Colorectal Cancer: A Review

**DOI:** 10.3390/biom14070874

**Published:** 2024-07-20

**Authors:** Qing Fang, Yunhua Xu, Xiangwen Tan, Xiaofeng Wu, Shuxiang Li, Jinyi Yuan, Xiguang Chen, Qiulin Huang, Kai Fu, Shuai Xiao

**Affiliations:** 1 Cancer Research Institute, The First Affiliated Hospital, Hengyang Medical School, University of South China, Hengyang 421001, China; q3145692607@163.com (Q.F.); xyh940707@163.com (Y.X.); txw1143122828@hotmail.com (X.T.); 15674919272@163.com (X.W.); 2 Department of Clinical Laboratory Medicine, The First Affiliated Hospital, Hengyang Medical School, University of South China, Hengyang 421001, China; 3 Institute of Clinical Medicine, The First Affiliated Hospital, Hengyang Medical School, University of South China, Hengyang 421001, China; 4 Department of Hepatobiliary Surgery, The Second Affiliated Hospital, Hengyang Medical School, University of South China, Hengyang 421001, China; 5 Department of Gastrointestinal Surgery, The First Affiliated Hospital, Hengyang Medical School, University of South China, Hengyang 421001, China; t17872114789@163.com (S.L.); 18075933997@163.com (J.Y.); cxgxhhncn@126.com (X.C.); 2018011993@usc.edu.cn (Q.H.); 6 Institute of Molecular Precision Medicine and Hunan Key Laboratory of Molecular Precision Medicine, Xiangya Hospital, Central South University, Changsha 410008, China

**Keywords:** pyroptosis, colorectal cancer, proliferation, growth, therapy

## Abstract

Colorectal cancer (CRC) is one of the leading causes of cancer-related mortality worldwide. The unlimited proliferation of tumor cells is one of the key features resulting in the malignant development and progression of CRC. Consequently, understanding the potential proliferation and growth molecular mechanisms and developing effective therapeutic strategies have become key in CRC treatment. Pyroptosis is an emerging type of regulated cell death (RCD) that has a significant role in cells proliferation and growth. For the last few years, numerous studies have indicated a close correlation between pyroptosis and the occurrence, progression, and treatment of many malignancies, including CRC. The development of effective therapeutic strategies to inhibit tumor growth and proliferation has become a key area in CRC treatment. Thus, this review mainly summarized the different pyroptosis pathways and mechanisms, the anti-tumor (tumor suppressor) and protective roles of pyroptosis in CRC, and the clinical and prognostic value of pyroptosis in CRC, which may contribute to exploring new therapeutic strategies for CRC.

## 1. Introduction

Colorectal cancer (CRC) is one of the most frequently occurring and the second leading cause of tumor mortality worldwide [1,2]. CRC is considered as a multifactorial disorder [3] whose development and progression are associated with age, ethnicity, lifestyle, abnormal activation of proto-oncogenes, and inactivation of tumor suppressor genes, etc. [4,5]. The malignant proliferation of tumor cells is a fundamental feature of cancer occurrence, progression, and metastasis, including CRC [6,7]. The proliferation and growth of tumors are closely associated with various cellular and biological processes, particularly the aberration of regulated cell death (RCD). As a result, a thorough knowledge of these molecular mechanisms and the inhibition of the proliferation of tumor cells have become a primary focus in cancer therapy, including CRC treatment.

Pyroptosis is a programmed cell death (PCD) executed by the gasdermin (GSDM) protein family [8], which was first discovered in macrophages infected with *Salmonella* [9] or *Shigella flexneri* [10] in the 1990s. This form of cell death was initially misclassified as cell apoptosis, considering that both types of cell death are characterized by caspase-dependent cell death, DNA damage induction, and nuclear condensation [11,12]. In 2001, the death pattern caused by *Salmonella* infection with macrophages was formally defined as “pyroptosis” [13,14]. Several studies had found that GSDM is the pyroptosis executive protein that could be cleaved by caspase proteins. The N-terminal domain of GSDM (GSDM-N) perforates (punches holes in) the cell membrane after binding to the cell membrane lipid, eventually leading to the rupture of the cell membrane. After the rupture, the contents leak out, leading to cell death and inflammation [15]. In recent years, pyroptosis has been reported to be associated with various diseases, including inflammatory diseases [16], cardiovascular disease [17], leukemia [18], and especially cancer [19].

The pathogenesis of cancer is a complicated biological process which involves several cellular processes including inflammation and RCD like pyroptosis, among many others. Inflammation, a physiological process of the body in response to harmful stimuli, is proven to be a critical factor in cancer development and progression. Pyroptosis is a specific form of cell death, which is also present in the context of inflammation. Numerous studies have shown that pyroptosis and relevant molecules are closely associated with the occurrence, development, and prognosis of melanoma, breast, gastric, liver, and lung cancer, as well as the CRC [20,21,22,23,24,25,26]. Pyroptosis plays a significant tumor suppressor function in melanoma and an important role in treatment [19,27]. However, cell pyroptosis in CRC appears to be a double-edged sword [28,29,30]. While on the one hand, the excessive release of inflammatory mediators, such as interleukin-1β (IL-1β) and IL-18, trigger abnormal systemic inflammatory reactions, accelerating tumor progression and increasing tumor burden, on the other hand, it promotes immunogenic cell death, enhances immune activity, and selectively kills tumor cells, exhibiting potential anti-tumor activity [31,32]. This review comprehensively analyzed the interplay between pyroptosis and CRC development, progression and clinical significance, which may contribute to exploring new therapeutic strategies for CRC. 

## 2. Molecular Mechanisms of Pyroptosis and CRC

**Figure 1:**
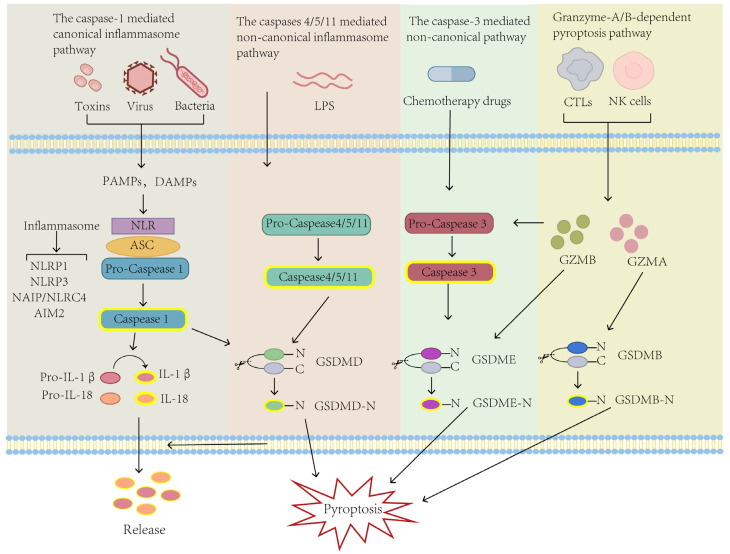
The molecular mechanism of pyroptosis. In the caspase-1 mediated canonical inflammasome pathway, after recognition by PRRs, PAMPs form inflammasome complexes. The inflammasome recruits and binds with ASC, activating caspase-1, which induces the formation of perforation-active GSDMD-N. GSDMD-N is released and forms pores in the plasma membrane, leading to secretion of IL-18/1β, influx of water, cell swelling, and rupture, ultimately resulting in pyroptosis. On the other hand, the non-canonical inflammasome pathway of pyroptosis involves recognizing LPS and activating caspase-4/5/11, which cleaves GSDMD to translocate to the cell membrane, triggering pyroptosis. Another novel non-canonical of pyroptosis mediated by caspase-3 is regulated through GSDME. Chemotherapeutic drugs activate caspase-3 to cleave GSDME, leading to widespread pyroptosis. Additionally, in the granzyme-dependent pathway of pyroptosis, GZMB and GZMA can respectively act on GSDME and GSDMB to induce pyroptosis. PAMPs: pathogen-associated molecular patterns; DAMPs: damage-associated molecular patterns; NLRP1: nucleotide-binding domain leucine-rich repeat pyrin domain containing 1; NLRP3: nucleotide-binding domain leucine-rich repeat pyrin domain containing 3; NAIP: NOD-like receptor family apoptosis inhibitory protein; NLRC4: NLR-family CARD-containing protein 4; AIM2: Absent in melanoma 2; ASC: Apoptosis-associated speck like protein containing a CARD; LPS: Lipopolysaccharide.

Pyroptosis is an intricate molecular biological process. Four pyroptosis-related molecular pathways have been identified, namely, the inflammasome-dependent canonical or non-canonical pathway, the caspase-3 mediated non-canonical pathway, and the granzymes-based pathway, all regulated by GSDMs (Figure 1).

### 2.1. The Caspase-1-Mediated Canonical Inflammasome Pathway

The first identified pathway of pyroptosis is the canonical inflammasome pathway. The canonical inflammasome pathway is caspase-1 dependent, and the inflammasomes including NLRP1 (nucleotide-binding domain leucine-rich repeat pyrin domain containing 1), NLRP3 (nucleotide-binding domain leucine-rich repeat pyrin domain containing 3), NAIP (NOD-like receptor family apoptosis inhibitory protein)/NLRC4 (NLR-family CARD-containing protein 4), and AIM2 (Absent in melanoma 2) are the key components of this pyroptosis pathway [33,34,35]. In this pathway, inflammasome activation is observed. After the stimulation by bacteria, virus, or intracellular danger signal, distinct types of pattern recognition receptors (PRRs) act as intracellular molecular sensors to bind and activate caspase-1 via apoptosis-associated speck-like protein containing a CARD (ASC), which forms the inflammasome [36]. After the inflammasome is successfully assembled, the precursor caspase-1 is cleaved into active caspase-1. Activated caspase-1 then cleaves GSDMD to form an active N-terminal domain of GSDMD (GSDMD-N) that induces cell membrane perforation, resulting in cell rupture, death, content release, and inflammation [37]. Meanwhile, caspase-1 promotes the maturation and secretion of IL-1β and IL-18 outside the cells, recruits more inflammatory cells, enhances the inflammatory response, and increases osmotic pressure and cytolysis [38]. 

The advantageous role of the canonical inflammasome pathway of pyroptosis in CRC has been confirmed in many studies [39,40]. Secoisolariciresinol (SECO) diglucoside (SDG) is a component of lignans with biological and anti-tumor activity [41]. In CRC, SDG enhances cleavage of the N-terminal domain of GSDMD (GSDMD-N) and caspase-1 in CRC cells [42]. A study found that in a cell model with FOXP2 depletion, low expression of FOXP2 promoted the cell growth of CRC and inhibited cell pyroptosis by inhibiting caspase-1 expression [43]. Another study found that ginsenoside Rh3 (GRh3) isolated from Chinese herbal medicine inhibited CRC cell proliferation and activated caspase-1 to induce GSDMD-dependent cell pyroptosis [44]. Also, subunit CLNA1 of *Lactobacillus plantarum* ZS2058 activates caspase-1, inducing pyroptosis in CRC cells, while subunit CLNA2 inhibits CRC cell proliferation by activating caspase-4/-5 [45]. Above all, the canonical inflammasome pathway in CRC is characterized by the formation of inflammasomes and activation of caspases, as well as subsequent cleavage of GSDMD, resulting in cell pyroptosis and inflammatory responses which finally lead to tumor suppression [8].

### 2.2. The Caspase-4/5/11 Mediated Non-Canonical Inflammasome Pathway

The non-canonical inflammasome pathway is relatively rare compared to the canonical one and occurs independent of caspase-1 activation [46]. The activation of the non-canonical inflammasome pathway is mediated by human caspase-4/5 and mice caspase-11. Studies have found that caspase-4/5 in humans or caspase-11 in mice directly binds to LPS and is activated under stress. Activated caspase-4/5/11 cleave GSDMD to form the activated N-terminal domain of GSDMD (GSDMD-N) with a perforation function, which causes cell membrane perforation and secretion of IL-1β and IL-18, resulting in pyroptosis [47,48]. According to reports, caspase-11 has been found to exert preventive and protective effects against dextran sodium sulfate (DSS)-induced colitis in mice [49]. Mice lacking caspase-11 exhibit prominent inflammatory responses and decreased proliferation of intestinal epithelial cells [50,51]. Therefore, in the non-canonical inflammasome pathway, the inhibitory effect of pyroptosis on CRC cells is also evident.

### 2.3. The Caspase-3 Mediated Non-Canonical Pathway

In addition to the inflammatory caspase-1/4/5/11, other apoptosis-associated caspases can also trigger pyroptosis. For example, under specific circumstances, such as chemotherapy or targeted therapy, pyroptosis can be induced by caspase-3 mediated gasdermin E (GSDME) [52]. This mode of pyroptosis is mediated by GSDME instead of GSDMD. Lobaplatin, a common chemotherapy drug, was reported to cause CRC cells to undergo pyroptosis by triggering caspase-3 to cleave GSDME [53]. Also, lobaplatin can activate caspase-3 in CRC cell lines HT-29 and HCT-116, leading to the cleavage of GSDME and subsequent occurrence of pyroptosis-like features, such as plasma membrane swelling and pore formation in CRC cells and inhibiting tumor growth [53]. The chemotherapeutic drug doxorubicin (DOX) can also induce the caspase-3 mediated pyroptosis through the ROS/JNK signaling pathway [54]. 

In addition to chemotherapeutic drugs, natural compounds can induce pyroptosis in many cancers. Neobractatin (NBT), isolated from the edible fruit of *Garcinia bracteata*, can induce GSDME cleavage by activating caspase-3 in esophageal cancer (EC) cells, resulting in cell death and tumor growth inhibition [55]. Apoptin possesses the ability to cause cell death in certain human cancer cell lines [56]. Apoptin from the *VP3* gene of the chicken anemia virus can also trigger GSDME-mediated pyroptosis by cleaving caspase-3 [56]. In addition, it can increase internal reactive oxygen species (ROS) while inhibiting HCT-116 cell viability and induce pyroptosis in nude mice bearing HCT-116 xenograft, inhibiting tumor growth [57]. 

### 2.4. Granzyme-A/B-Dependent Pyroptosis Pathway

According to recent research, granzymes can also target tumor cells via perforin and trigger pyroptosis [58,59]. Granzyme is a serine protease secreted by cytotoxic T lymphocytes (CTL) and natural killer (NK) cells [58,60]. Five kinds of granzymes have been identified in humans: GZMA, GZMB, GZMH, GZMK, and GZMM [58]. A previous study found that GZMA cleaves GSDMB, resulting in pyroptosis of SW837 and SKCO1 cells [59]. In CT26 mouse cells, the cleavage of GSDMB by GZMA significantly promotes tumor clearance [58]. Another serine protease, GZMB, can directly cleave GSDME and trigger caspase-independent pyroptosis in HEK293T cells. Increased expression of GSDME enhances the phagocytic activity against tumor cells, thereby inhibiting tumor growth [59].

## 3. GSDM Family and CRC

The GSDM protein family is characterized by pore formation, mainly expressed in the digestive tract, skin, and immune cells [61]. According to the conserved C-terminal and N-terminal domain sequences, the GSDM family is divided into GSDMA, GSDMB, GSDMC, GSDMD, and GSDME, except pejvakin (DFNB59/PJVK) [62]. GSDMs can regulate normal cell proliferation and differentiation, protect the host from pathogens [25], and act as an effector protein for pyroptosis to trigger inflammation and cell death [63,64]. GSDMs have been associated with various human diseases, including cancer and inflammation-associated diseases. Previous studies found that the GSDM family proteins are expressed in healthy normal tissues but also highly expressed in cancer tissues [65]. Recently, the GSDM family’s role in cancer has become more prominent. The abnormal expression and dysfunction of GSDM family genes were linked to multiple cancer-related pathways, suggesting that GSDM genes are extensively involved in cancer occurrence and progression, including breast cancer [54], lung cancer [66], and gastric cancer [24], as well as in CRC [67]. Furthermore, the GSDM genes showed significant genomic alterations, according to pan-cancer studies of the GSDM family [65]. 

**Figure 2:**
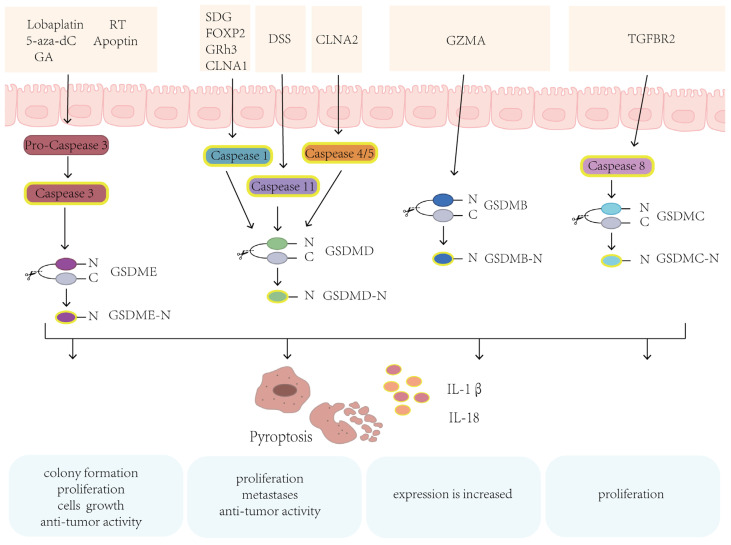
Signaling pathways of GSDMs in the CRC. GSDM proteins can regulate cell proliferation and differentiation and also serve as effector proteins in pyroptosis, triggering inflammation and cell death. Various stimuli and upstream signals such as lobaplatin, RT, 5-aza-dC, apoptin, and GA can induce GSDME cleavage and subsequent cell death. Caspase-1/GSDMD-dependent pyroptosis is induced by activating molecules, including SDG, FOXP2, GRh3, and CLNA1. CLNA2 induces cell pyroptosis through the activation of caspase-4/5. GSDMB and GSDMC participate in pyroptosis and subsequent cell death through the GZMA and TGFBR2, respectively. 5-aza-dC: 5-aza-2′-deoxycytidine; RT: radiation therapy; GA: gambogic acid; DSS: dextran sulfate sodium; GRh3: ginsenoside Rh3.

According to several studies, certain medications or molecules can cause GSDM-mediated pyroptosis in CRC, suggesting that GSDM family genes were associated with the development and progression of CRC [68]. Notably, CRC patients with low expression of GSDMD have shown poor prognosis [69]. According to the Venn analysis, GSDMB, GSDMD, and GSDME are related to the invasion and metastasis of CRC [65], suggesting that GSDM protein family-mediated pyroptosis has an important role in CRC (Figure 2, Table 1).

### 3.1. GSDME

GSDME, also known as deafness autosomal dominant 5 (DFNA5), can induce cell swelling and death [75,76]. After cleavage by the activated caspase-3, the N-terminal domain of GSDME (GSDME-N) can promote pore formation and trigger pyroptosis. GSDME expression level is different in malignant and normal tissues [77]. GSDME is always highly expressed in normal tissues, but the expression level is distinct among different cancers and episodically absent expression in some cancer, and the absent expression may be due to the methylation of the GSDME gene promoter [67,78,79,80]. The mutations of tumor-associated GSDME also could inhibit pyroptosis [81].

In addition, several studies have shown that GSDME, as a molecule with known anti-tumor potential, is involved in the development and progression of many cancers [82]. According to a recent study, ectopic expression of GSDME in cancer cells could enhance anti-tumor immune responses and suppress tumor growth [59]. In case of CRC also, GSDME has been shown to have anti-tumor effect [83]. For example, an ectopic expression of GSDME significantly inhibits colony formation, proliferation, and growth of CRC cells [73]. The methylation inhibitor 5-aza-2′-deoxycytidine (5-aza-dC) could promote GSDME expression and inhibit tumor cell proliferation and carcinogenesis, implying that GSDME is a potential tumor suppressor gene in CRC [73,84]. Radiation therapy (RT) and chemotherapeutic agents such as lobaplatin and cisplatin can also induce caspase-3 activation and GSMDE cleavage following by pyroptosis [53,85]. Activated caspase-3 and cleavage of GSDME were also observed in natural compound gambogic acid (GA)-treated CRC cells. This GA induced GSDME-dependent pyroptosis and significantly suppressed tumor proliferation, while enhancing anti-tumor activity [86]. Since GZMB can cleave and activate caspase-3, Zhang et al. also found that GZMB cleaved and activated GSDME in a caspase-independent manner [59]. These findings indicate that GSDME induces pyroptosis in CRC, acting as a cancer suppressor gene.

### 3.2. GSDMD

GSDMD is the extensively studied member of the GSDM family, being generally recognized as the executor of pyroptosis [8,47]. GSDMD is mainly expressed in the gastrointestinal epithelium, skin, and immune cells [87,88]. GSDMD is significantly expressed in esophageal cancer, gastric cancer, CRC, and other cancers [87,89]. Typically, inflammatory caspase-1, which was activated by different signals/stimuli, cleaves the GSDMD to form an activated N-terminal domain of GSDMD (GSDMD-N), leading membrane pores to induce pyroptosis, which mediates the release of inflammatory cytokines IL-1β and IL-18 [90,91,92]. During pyroptosis, the calcium influx through the GSDMD pores is one of the signals to activate cell membrane repair, which enhances cell survival ability [93]. In addition to caspase-1, caspase-11 and caspase-4/5 can also cleave GSDMD and induce pyroptosis. Caspase-11 and caspase-4/5 can be directly activated by LPS to cleave GSDMD and induce pyroptosis [8,47]. Pan-cancer analysis showed that GSDMD-mediated pyroptosis might have a critical role in cancers such as adrenocortical carcinoma and CRC and is associated with the prognosis [94]. In a GSDMD deficient CRC mouse model induced by azoxymethane (AOM)/dextran sulfate sodium (DSS), CRC development is accompanied by the downregulation of GSDMC [95]. Also, a study found that a cooperative down-regulated expression of GSDMD and GSDMC had a suppressive effect on gastrointestinal tumors [95]. Furthermore, GSDMD expression seems to be negatively correlated with lymphatic metastases and distant metastases in CRC [96]. According to another study, LPS-induced pyroptosis inhibits the proliferation of CRC and increases the anti-tumor activity of oxaliplatin [69]. Pyroptosis induced by caspase-4/GSDMD can also promote the release of inflammatory factors such as IL-1β and IL-18, as well as recruit CD8^+^ T cell infiltration, thereby activating anti-tumor immunity [97]. Collectively, GSDMD-mediated pyroptosis has an important anti-tumor role in CRC.

### 3.3. GSDMB

GSDMB is known as gasdermin-like protein (GSDML). Interestingly, GSDMB is unique to the human genome and particularly expressed in the airway tract and gastrointestinal epithelium [87,98]. Furthermore, it is abnormally expressed in many malignancies, including breast, cervical, gastric, and colon cancers [22,87,99]. GSDMB is frequently up-regulated and has a complex role in cancer. Additionally, GSDMB expression is increased in CRC and associated with inflammatory bowel disease (IBD) susceptibility. Therefore, GSDMB may have an important role in the pathogenesis of CRC under the inflammatory response in IBD [100,101]. 

A GSDMB-dependent cellular function study suggested that GSDMB-expressing intestinal epithelial cells (IECs) have a protective effect during gastrointestinal inflammatory infection and cancer [71,102]. Recent studies have found that GSDMB could inhibit epithelial cell proliferation and motility, suggesting that epithelial-derived GSDMB has a protective function from cancer [70]. In addition, GSDMB has been found to specifically bind with lipid membranes, but this property appeared to be independent of pyroptosis [70,102]. Since GSDMB has no orthologs in mice, identifying its role in vivo is still challenging. At present, the evidence related to the molecular mechanism of GSDMB-induced pyroptosis is limited. Therefore, the important role of GSDMB in cancer cell pyroptosis remains elusive.

### 3.4. GSDMC

GSDMC was originally found to be highly expressed in metastatic melanoma cells and was first known as a melanoma-derived leucine zipper-containing extranuclear factor (MLZE) [84]. The expression of GSDMC is low in most normal tissues but high in the gastrointestinal tract. Also, its physiological function is still unclear [87]. In most cancers, the methylation of the GSDMC promoter is lower in tumors than in normal tissues. GSDMC expression is tumor-specific and often expressed in melanoma [84], lung cancer [103], and CRC [104]. In addition, GSDMC exhibits cell growth inhibition activity, which indicates it may act as a potential tumor suppressor [87]. Like other GSDMs, GSDMC is involved in the pyroptosis biological process. Yet, because the biological function of GSDMC is rarely studied, its role in cancer is still not fully understood. 

Hou et al. reported that GSDMC could be cleaved by caspase-8 and cause breast cancer cells to undergo pyroptosis [105]. Another study discovered that the metabolite α-ketoglutarate (α-KG) could induce pyroptosis via caspase-8-mediated GSDMC cleavage, inhibiting tumor growth and metastasis in a mouse model [106]. GSDMC also inhibits cell growth and decreases tumor proliferation in gastric and esophageal cancer [87]. On the other hand, GSDMC has also been linked to tumorigenesis in some cases. For example, GSDMC stimulates CRC proliferation under adenomatous polyposis coli (APC) mutation, which was significantly reversed by GSDMC silencing [23]. However, due to various pathways of pyroptosis, the tumor suppressor or tumor-promoting effect of pyroptosis is also related to the duration time and the intensity of the inflammatory response. Therefore, the function of GSDMC in the carcinogenesis of CRC needs to be further clarified.

### 3.5. Other GSDMs

#### 3.5.1. GSDMA

GSDMA is a GSDM family member with three mouse homologs [62]. The molecular regulation mechanism of GSDMA is still not fully understood. GSDMA is expressed in the upper gastrointestinal tract and is strongly linked with asthma susceptibility [107], but its expression is frequently silenced in gastric cancer, suggesting the potential tumor suppressor role in gastrointestinal cancer [87]. However, since GSDMA is not expressed in the small and large intestine, the correlation of GSDMA with CRC development or progression has not been identified.

#### 3.5.2. DFNB59

DFNB59, also known as Pejvakin (PJVK), has received little attention as a member of the GSDM family so far. Its expression has been found in the neurons of the afferent auditory pathway, inner ear cells, and testis, and it is mainly associated with hearing impairment [108,109]. DFNB59 lacks the linker region between the N-terminal and C-terminal domains and has no pore-forming activity [110]; thus, its role in pyroptosis is still unknown. The role of DFNB59 in CRC has not yet been reported.

## 4. The Role of Pyroptosis in CRC

### 4.1. Pyroptosis Function as a Tumor Suppressor in CRC

Emerging studies demonstrate that various cellular processes and RCD may lead to cancer cell death, and the most common are apoptosis, autophagy, ferroptosis, necroptosis, and pyroptosis. RCD abnormalities are associated with cancer development and progression [68,111,112]. Pyroptosis, an inflammatory cell death associated with the inflammasomes and GSDM, affects the transformation and development of cancer. The activation of pyroptosis can lead to selective cell death in tumor cells. Cancer cells exhibit a higher sensitivity to pyroptosis compared to normal cells [113,114]. What is more, studies have proven that pyroptosis can promote immunogenic cell death, improve immune activity, and kill tumor cells, thus having a strong anti-tumor activity against cancer cell invasion [14,115,116]. A study found that FOXP2 could inhibit CRC cell proliferation by promoting caspase-1 expression in a colitis-associated CRC model [43]. In addition, using a bioorthogonal system, it was found that in tumor cells undergoing pyroptosis, cells and genes associated with immunity and anti-tumor, such as CD4^+^ T, CD8^+^ T, and NK cells, are up-regulated, while various molecules that promote tumor growth and proliferation are down-regulated [117]. Pyroptosis-related participants, pathways, and regulatory mechanisms have been implicated in cancer development, progression, and metastasis, and pyroptosis has a powerful ant-tumor potential during cancer progression, including CRC [58,59]. Therefore, the activation of pyroptosis may provide a potential tumor suppressor and treatment strategy for CRC.

### 4.2. Pyroptosis and the Tumorigenesis and Development of CRC

Various factors regulate the pathophysiological process of tumorigenesis. The two primary processes underlying CRC occurrence are the classical normal mucosal–adenoma–carcinoma evolution pattern and the colitis-associated pattern of CRC [5]. Pyroptosis takes part in most aspects of the tumorigenesis and development of CRC [118,119,120]. According to a classical pattern, CRC originates from cancer stem cells (CSC). Previous studies indicated that pyroptosis could decrease CSC activation to inhibit cancer cell proliferation. After evaluating the relationship between GSDM genes and stemness, it was found that the GSDM family was not only related to stemness but also to patients’ survival [65]. The relationship between the normal mucosal–adenoma–carcinoma evolution pattern and pyroptosis remains unclear and requires further exploration and investigation. However, the relationship between pyroptosis and colitis-associated CRC is gradually becoming clearer. 

For the inflammation-related pattern, IBD is highly associated with CRC and can be divided into Crohn’s disease (CD) and ulcerative colitis (UC) [121]. During the inflammatory process, PAMPs from pathogens and DAMPs released by host damaged cells are recognized by PRRs in intestinal epithelial cells, leading to inflammasome assembly and subsequent cell pyroptosis. Accordingly, pyroptosis promotes cell death via the inflammatory response and protects from CRC. The anti-tumor role of inflammasomes in colitis-associated CRC such as maintaining epithelial cell integrity by NLRP3 inflammasomes in IBD has been extensively studied. Several studies have identified the anti-tumor effects of pyroptosis in CRC [18,122,123]. It has been found that NLRP1^−/−^ mice had significantly increased gastrointestinal inflammation and tumorigenesis compared with wild-type mice, indicating that NLRP1 has a protective role in reducing colitis and colitis-related CRC [40]. Another study demonstrated that NLRP3 inflammasome components act as a protective role in an animal model of acute colitis, in which NLRP3 deficiency enhanced chemically induced colitis-associated CRC occurrence [122]. Meanwhile, in the acute intestinal inflammation model induced by dextran sodium sulfate (DSS), caspase-11 was found to suppress and inhibit intestinal inflammation, further supporting the protective role of pyroptosis in IBD [50]. In mice lacking caspase-11, the damage caused by inflammation was more pronounced in the colon [124]. Moreover, in the colitis-associated CRC model induced by 5-aza-dC, the activation of NALP1 inflammasome increased, suppressing the growth of colon cancer and increasing lifespan [114]. A study found that NLRP3 could promote hepatic NK cell maturation, and the liver metastasis was increased when IL-18 signaling was impaired in a NLRP3 knockdown CRC mouse model [125]. All the above findings suggest that pyroptosis plays an important role in CRC development and progression.

## 5. The Potential Clinical Value of Pyroptosis in CRC

**Figure 3:**
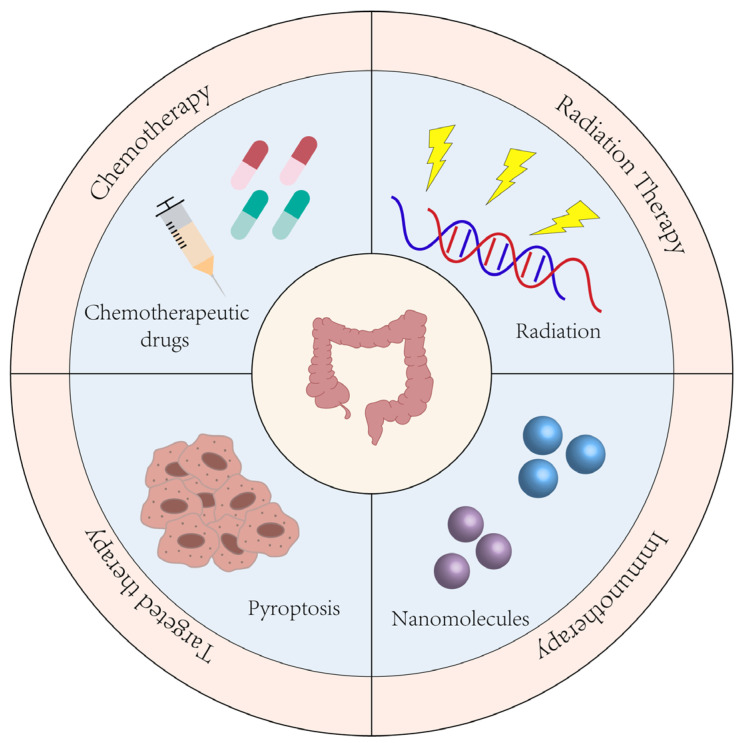
Prospects of pyroptosis in CRC treatment. The use of chemotherapy drugs, RT, immunotherapy, and targeted pyroptosis can effectively induce CRC cells pyroptosis, thereby improving the treatment effect.

According to the important role of pyroptosis in the development and progression of CRC, the significance of pyroptosis in CRC treatment has also been studied (Figure 3). Chemotherapy is currently the most widely utilized treatment for CRC. Chemotherapeutic drugs can induce pyroptosis in tumor cells, affecting cell vitality, invasion, and migration, thereby promoting tumor cell death [69,114]. Additionally, the synergy between pyroptosis and chemotherapy drugs can increase chemotherapy sensitivity. Radiotherapy-induced pyroptosis enhances the radiosensitivity of CRC and boosts tumor immune infiltration, greatly improving treatment efficacy [126,127]. In CRC immunotherapy, induction of cell pyroptosis promotes immune cell activity, enhances cancer cell sensitivity to immune checkpoint inhibitors (ICIs), and improves anti-tumor immune responses [58,117,128]. Furthermore, targeted pyroptosis may become an important therapeutic approach in future CRC treatments. Recently, novel delivery molecules such as biomimetic nanoparticles (BNP) and drug–polymer hybrid supramolecular nanoprodrugs (PDNP) have been developed, effectively inducing pyroptosis in tumor cells and enhancing treatment efficacy [129,130].

### 5.1. Pyroptosis and Chemotherapy in CRC

Chemotherapy is the most important systemic treatment option for advanced CRC. Yet, due to chemotherapy resistance, many CRC patients develop recurrence and metastasis after therapy [131]. Therefore, developing treatment strategies to reverse or compete against chemotherapy resistance is crucial. 

Some chemotherapeutic drugs, including cisplatin, paclitaxel, 5-fluorouracil (5-FU), lobaplatin, etc., can trigger pyroptosis in tumor cells [132]. Compared to conventional chemotherapy, drugs inducing pyroptosis can effectively overcome resistance to apoptosis, reduce tumor immune tolerance, and enhance treatment efficacy. In CRC with high expression of GSDME, drugs such as TNFα+CHX and navitoclax can induce pyroptosis through the BAK/BAX caspase-3-GSDME pathway [81]. However, due to hypermethylation of the GSDME gene, most cancer cells lack caspase-3-derived GSDME. Four inhibitors of cell proliferation, including obatoclax mesylate (OM), BI 2536 (BI), (S)-(+)-camptothecin (CPT), and bortezomib (BTZ), were screened for their drug efficacy in CRC using a cancer-tissue-originated spheroid (CTOS)-based screening method and successfully induced GSDME-mediated pyroptosis. The results showed that CRC growth was inhibited and accompanied by increased CD8^+^ T cells during pyroptosis [133]. More recently, Alisol A, a marine herb with anti-tumor effects, was found to induce pyroptosis by increasing the levels of caspase-1, GSDMD, and GSDME in CRC cells, while reducing cancer cell migration and increasing the chemosensitivity of cisplatin [134]. FL118 is a novel camptothecin-based anti-tumor drug that exhibits anti-tumor effects in CRC. Recent studies have found that FL118 inhibits the growth and metastasis of CRC by activating the NLRP3 inflammasome and promoting the release of IL-18 and IL-1β [135]. Additionally, it was found that administering the synthetic FXR agonist GW4064 simultaneously with oxaliplatin could have a synergistic anti-tumor impact, as GW4064 enhanced the chemosensitivity of cells to oxaliplatin by inducing BAX/caspase-3/GSDME-mediated pyroptosis [136]. Similarly, a study showed that GSDMD-mediated pyroptosis promoted oxaliplatin sensitivity in HT-29 cells after LPS-induced GSDMD expression [69]. This combination method provided a new strategy for the treatment of CRC.

In conclusion, all this evidence suggests that the induction of pyroptosis by chemotherapeutic drugs could promote drug sensitivity and improve the efficacy of chemotherapy.

### 5.2. Pyroptosis and RT in CRC

RT is one of the fundamental therapies to control local recurrence and metastasis in advanced CRC. Several research investigations have shown that the induction of cancer cell pyroptosis is also an important mechanism of RT [126,137]. Studies found that RT could effectively induce dose-dependent pyroptosis in HCT-116 cells [127]. In CRC models, RT suppressed the expression of miR-15a while inducing caspase-1 activity and GSDMD expression, decreasing tumor cell vitality, proliferation, and growth [137]. Additionally, caspase-1, caspase-4, and caspase-5 were up-regulated and triggered pyroptosis as a result of miR-21-5p overexpression [138]. Another study showed that the lncRNA NEAT1 regulates the expression of GSDME, leading to pyroptosis in CRC cells during RT [127]. Elevated expression of GSDME enhances the RT sensitivity of CRC and increases tumor immune infiltration [126,127]. Thus, the induction of pyroptosis via RT is important for enhancing the RT efficiency in controlling tumor growth and immune microenvironment in CRC.

### 5.3. Pyroptosis and Immunotherapy

The fully functional immune system can prevent tumor initiation and progression, but the immune function is always impaired under malignant progression [139,140]. Immunotherapy is a treatment strategy to enhance the cancer cell immunogenicity and stimulate the immune response against immune escape [141]. Immunotherapy based on ICIs significantly affects most cancers, especially CRC. In fact, the application of ICIs is limited because of their natural anti-apoptotic ability and the individual differences in patients. Thus, studying the molecular mechanisms of cell death other than apoptosis has become a new subject of cancer therapy [142]. 

Infiltration of CD8^+^ T cells is crucial to immunotherapy. Activated T cells can exert synergistic effects in inducing pyroptosis and immune response [142]. During pyroptosis, the immune cells’ activity is enhanced, leading to an increased cytotoxic effect of cytotoxic lymphocytes [58]. Therefore, induction of tumor cell pyroptosis can improve the sensitivity to ICIs. For instance, nanomaterials enhanced the immunogenicity of cancer cells and showed favorable treatment efficacy in immunogenic cell death (ICD)-based therapy [143]. In addition, ICIs and pyroptosis were also found to show synergistic anti-tumor activity [117,128]. A prodrug utilizing high paclitaxel (PTX) and a photosensitizer purine 18 (P18)-loaded ROS/glutathione (GSH) dual-responsive nano-prodrug (denoted as MCPP) was recently designed, which could efficiently induce cancer cell-specific pyroptosis, trigger adaptive immunity, enhance the effectiveness of immune checkpoint blockade (ICB), and achieve tumor regression [144]. These findings suggest that pyroptosis is essential for CRC immunotherapy.

### 5.4. Pyroptosis and Targeted Therapy 

Distinct signaling pathways and molecules of novel cell death forms other than apoptosis have been discovered recently, providing new targets and strategies for cancer therapy [145]. As an emerging target for cancer therapy, pyroptosis has shown great potential in treating CRC [127,146]. A series of new drugs targeting pyroptosis are being developed and tested in clinical trials in CRC. For example, a biomimetic nanoparticle (BNP) could efficiently accumulate at tumor sites, induce GSDME-mediated pyroptosis, and activate systemic anti-tumor immunity, effectively reducing the severe toxicity to normal cells and tissues [129]. A nanoparticle-mediated cytotoxic drug can selectively be delivered to cancer cells via exotoxin A to activate NLRP3, cleave GSDMD, and mediate pyroptosis at the primary tumor site [72]. Moreover, nanoparticles with high selectivity for inducing pyroptosis can effectively activate caspase-11 and NLRP3, causing CRC cell proliferation suppression. This treatment’s maximum effect appears 48 h post-treatment [147]. Moreover, Liang and colleagues designed a drug–polymer hybrid supramolecular nanoprodrug (PDNP) as a pyroptosis inducer. PDNP solves the weakness of pyroptotic efficacy during drug delivery and promotes precise drug release, thereby effectively triggering GSDME-mediated pyroptosis and enhancing the anti-tumor immune response [130]. These findings provide a lot of preliminary targeting pyroptosis therapy evidence for CRC.

### 5.5. Diagnostic and Prognostic Value of Pyroptosis in CRC

As the important role of pyroptosis in CRC has been identified, the diagnostic and prognostic value of pyroptosis in CRC has also gradually been studied in recent years. Several pyroptosis-related biomarkers have been identified to predict patient prognosis [148,149]. For example, a pyroptosis-related gene (PRG) prognosis prediction model showed that pyroptosis is associated with a higher proportion of immune infiltration and indicated a better prognosis [150,151]. A study demonstrated that the expression of NLRC3 and NLRP4 is lowered in CRC and correlated with immune infiltration, which was positively correlated with favorable prognosis [120]. In a recent study, functional analysis revealed a negative correlation between three genes (SLC2A3, TMPRSS11E, and UPK3B) can predict the overall survival of patients with colon cancer, finding that it is negatively correlated with the proliferation and migration of colon cancer cells [152]. Moreover, the lncRNA risk model related to cell pyroptosis revealed remarkable predictive capacity in CRC, indicating that the high-risk group was worse than the low-risk group in terms of survival outcomes [153]. The role of the predictively modeled lncRNA KCNQ1OT in CRC progression has been reported [153]. In addition, a CRC risk model based on nine high-risk groups of lncRNA associated with pyroptosis has been validated, demonstrating high expression levels in cancer tissues [154]. It was found that pyroptosis levels in CRC (such as CSP1, CASP6, GZMB, and NLRP1) and the tumor microenvironment (TME) were significantly correlated with the prognosis of individual CRC patients [155]. The CRC prediction model based on 13 PRGs (AIM2, CASP1, CASP5, CASP6, CASP8, CASP9, ELANE, GPX4, GSDMD, NLRP7, NOD2, PJVK, and PRKACA) revealed a higher proportion of immune infiltration and better survival outcomes in the low-risk group [156]. In addition, IL-17A-mediated pyroptosis in CRC cells could release immune antigens and promote the infiltration of CD8^+^ T cells, thereby improving the CRC patients’ prognosis [97]. AIM2 inflammasome, which mediates caspase-1 activation and pyroptosis, had low expression in CRC and was negatively correlated with survival [157,158]. HMGB1 is an inflammatory nuclear protein released during GSDME-mediated pyroptosis, and in the absence of GSDME, the expression of HMGB1 is significantly reduced and associated with the prognosis of CRC [159]. 

The above studies indicate that pyroptosis plays an important function in CRC, and that pyroptosis-related molecules can be used as biomarkers for clinical diagnosis, treatment response, and prognosis prediction. 

## 6. Conclusions

Pyroptosis has emerged as a novel form of pro-inflammatory RCD, characterized by the GSDM-activated cellular lysis and subsequent release of cellular contents, triggering a potent inflammatory response, as well as an eventual cell death. Pyroptosis is closely associated with the development and progression of cancer, including CRC. A small number of studies suggest that pyroptosis may increase tumor burden [160,161]. Yet, most research has shown that pyroptosis has a significant anti-tumor effect in CRC. Consequently, pyroptosis is considered as a promising therapeutic strategy for CRC patients. Whether pyroptosis exerts its anti-tumor effects solely through known key activation components requires further investigation. The powerful therapeutic potential of inducing pyroptosis in CRC cells is apparent. It could overcome resistance to conventional chemotherapy drugs and synergize with immunotherapy to enhance anti-tumor immune responses, thereby improving treatment outcomes. The ongoing development of novel inducers targeting pyroptosis, such as biomimetic nanoparticles and supramolecular nanoprodrugs, broadens the therapeutic landscape of pyroptosis in CRC. In addition, numerous pyroptosis-related genes have emerged as valuable diagnostic prognostic and biomarker candidates in CRC. Overall, studies on pyroptosis in CRC will undoubtedly continue to expand, and a comprehensive understanding of the relationship between pyroptosis and CRC will furthermore bring new hope for preventing and treating CRC.

## Figures and Tables

**Table 1:** The expression and function of GSDM family members in CRC.

GSMD Family Gene	Role in CRC	Expression Pattern	Prognosis in CRC	References
GSDMA	Uncertain	Uncertain	Uncertain	None
GSDMB	Oncogene	Up-regulated	Negative	[70,71]
GSDMC	Oncogene	Up-regulated	Negative	[23]
GSDMD	Anti-oncogene	Down-regulated	Positive	[69,72]
GSDME	Anti-oncogene	Down-regulated	Positive	[67,73,74]
DFNB59/PJVK	Uncertain	Uncertain	Uncertain	None
